# Effect of prophylactic balloon occlusion of internal iliac artery in pregnancies complicated by placenta previa and accreta

**DOI:** 10.1186/s12884-021-04103-x

**Published:** 2021-09-21

**Authors:** Daijuan Chen, Jinfeng Xu, Yuan Tian, Pengfei Ye, Fumin Zhao, Xinghui Liu, Xiaodong Wang, Bing Peng

**Affiliations:** 1 Department of Obstetrics and Gynecology, Ministry of Education, West China Second University Hospital of Sichuan University/Key Laboratory of Birth Defects and Related Diseases of Women and Children (Sichuan University), No. 20, 3rd Section, South Renmin Road, Chengdu, 610041 Sichuan China; 2 Department of Radiology, West China Second University Hospital, Sichuan University, Chengdu, 610041 Sichuan China

**Keywords:** Interventional therapy, Internal iliac arteries, Placenta previa, Placenta accreta, Hemorrhage

## Abstract

**Background:**

Placenta previa and accreta are serious obstetric conditions that are associated with a high risk of intraoperative massive hemorrhage, the prophylactic intravascular balloon occlusion technique is increasingly used in managing uncontrolled hemorrhage in cesarean section (CS). We aim to examine the clinical effectiveness of prophylactic balloon occlusion of the internal iliac artery (PBOIIA) during CS in improving maternal outcomes for patients with placenta previa and accreta.

**Methods:**

A total of 420 women with placenta previa and accreta who underwent CS from January 2014 to December 2018 were included retrospectively. Patients were divided into balloon group in which patients had PBOIIA (*n* = 248) and the control group in which patients did not have PBOIIA (*n* = 172). Meanwhile, we performed a subgroup analysis in whether taking parallel transverse uterine incision (PTUI) surgery. Information on conditions of patients and newborns, perioperative blood indicators, surgical outcomes were collected.

**Results:**

Median estimated blood loss (mEBL) was 2200 mL in the balloon group and 2150 mL in the control group respectively, there was no significant difference between two-groups comparison (*P* > 0.05), and the rate of patients with hysterectomy was also has no difference between the two groups (36.3% verus 35.5%, *P* > 0.05), while there is a significant difference between two groups in the amount of PRBCs transfused [3 (0–31.5) verus 3 (0–39), *P* <0.05], moreover, the proportion of PRBCS> 8 units in the balloon group is significantly lower than that in control group (11.29% verus 23.26%, *P* <0.05).. However, the total hospitalization costs (45,624.4 ± 11,061.9 verus 37,523.1 ± 14,662.2, CYN) and surgery costs (19,910.6 ± 2622.6 verus 11,850.5 ± 3146.1, CYN) in balloon group were significantly higher than those in control group (*P* < 0.05). Subgroup analysis showed PTUI surgery had no significant differences in EBL (*P* >0.05), but it could significantly decrease hysterectomy rates (*P* <0.05).

**Conclusions:**

PBOIIA has no significant effect on reducing intraoperative EBL and hysterectomy rate in patients with placenta previa and accreta. and although it could reduce the intraoperative PRBCs in patients with massive hemorrhage, it significantly increases the financial cost for patients. Therefore, PBOIIA should not be routinely recommended to patients with placenta previa and accreta.

## Background

Placenta previa and accreta associated with previous cesarean section are related to severe adverse maternal–fetal pregnancy outcomes and are even accountable for a high risk of maternal death [1]. These placental conditions can cause disseminated intravascular coagulation, shock, and a high rate of hysterectomy [2]. Placenta previa and placenta accreta are the major causes of postpartum hemorrhage, and is currently a leading cause of maternal death worldwide [3]. Massive hemorrhage during cesarean section (CS), which is hard to predict and control, is the massive threaten to the life of patients with placenta previa and accreta. Cesarean hysterectomy is an important treatment for placenta previa and accreta, but it should be performed with caution [4]. Recently, a lot of conservative management has been conducted to reduce intraoperative hemorrhage and the hysterectomy rate, and to ensure maternal and newborn safety during CS. The prophylactic intravascular balloon occlusion technique is increasingly used in managing uncontrolled hemorrhage in CS. This technique includes prophylactic intravascular balloon occlusion of the internal iliac arteries (PBOIIA) and abdominal aorta arteries (PBOAA) [5].

Owing to the low risk of vascular complications [6], PBOIIA has been used in our hospital for patients with placenta previa and accreta since 2014. The effectiveness of PBOIIA remains controversial because of the inconsistent results of different research. Fan, Zhou et al. reported that PBOIIA was an effective method of hemostasis in CS. However, Feng, Salim, Chen et al. showed that PBOIIA had no benefit in reducing estimated blood loss (EBL) and improving maternal outcomes for patients with placenta previa and accreta. There is still a lack of large-sample studies on the effectiveness of internal iliac artery balloons. Therefore, we performed a large-sample, retrospective cohort study and aimed to evaluate the effectiveness and practicality of PBOIIA in improving maternal outcomes for patients with placenta previa and accreta.

## Methods

### Study population

This study was conducted from January 2014 to December 2018. The retrospective, observational study was approved by the Ethics Review Committee of West China Second University Hospital of Sichuan University. And because of the nature of the retrospective, observational setting for our study, and the data are anonymous, an informed consent was waived by the Ethics Review Committee of West China Second University Hospital. All study methods of the retrospective study were conducted following the relevant regulations of a protocol, which were approved by the Institutional Review Board from the West China Second University Hospital of Sichuan University.

Placenta previa occurred when the placenta was wholly or partially implanted in the lower uterine segment. Placenta accreta was defined as the situation where the placental trophoblast invaded into the myometrium, according to the depth of villous tissue invasiveness. Placenta accreta has been subdivided by modern pathologists into “creta” or “adherenta”. An adherent placenta is where the villi adhere superficially to the myometrium without interposing the decidua. Placenta increta is where the villi penetrate deeply into the uterine myometrium down to the serosa. Placenta percreta is where the villous tissue perforates through the entire uterine wall and may invade the surrounding pelvic organs, such as the bladder [7, 8].

All included patients had at least one prior CS and were diagnosed with placenta previa or placenta accreta when the placenta covered a previous cesarean scar, which was examined by color Doppler ultrasonography and pelvic magnetic resonance imaging (MRI) examinations before delivery. The diagnosis was confirmed by intraoperative findings or histopathological examination after surgery. Patients with an adherent placenta were excluded in this study, because balloon occlusion was not routinely used for these patients in our clinical work. Therefore, we only included patients with placenta increta or percreta. Patients with serious medical and surgical diseases (mainly include heart disease, pancreatitis and severe hepatitis, liver and kidney dysfunction, tumor, severe infectious diseases, preeclampsia, etc.), incomplete data, multiple pregnancies, or those who delivered before 28 weeks of gestation were excluded.

**Fig. 1:**
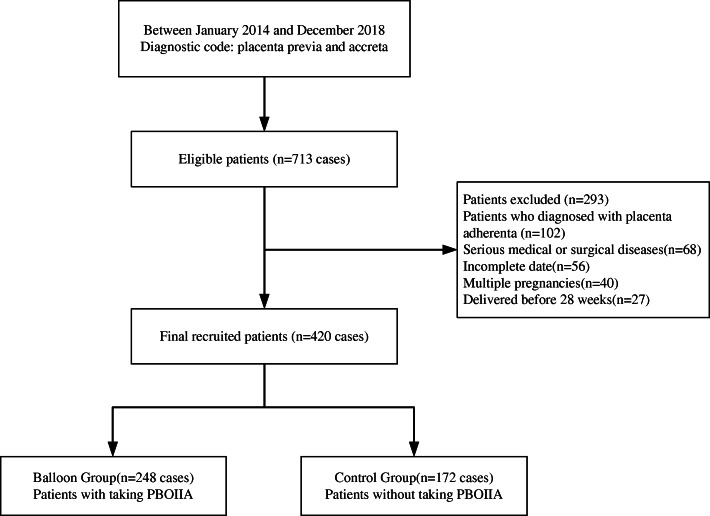
Flowchart of selection of the patients for the study. PBOIIA:prophylactic interventional therapy of the internal iliac artery balloon occlusion

Of 713 patients with placenta previa and accreta associated with previous CS who delivered in our hospital, 420 were included in this study finally (Fig. 1). The 420 patients were divided into two groups according to whether they had PBOIIA (balloon group) (*n* = 248, 59.0%) and whether they did not have PBOIIA (control group) (*n* = 172, 41.0%). In 2017, doctors in our hospital investigated a novel approach called parallel transverse uterine incision (PTUI) surgery. PTUI had a significant effect on reducing intraoperative blood loss and the hysterectomy rate for patients with placenta previa and accreta [9, 10]. Among our included patients, we found that some patients underwent PTUI surgery simultaneously. To avoid the effect of PTUI surgery on the results, we conducted a subgroup analysis on the two groups of patients according to whether they had PTUI surgery during CS. Among the 420 patients, 86 had PTUI surgery and 334 did not have PTUI surgery. In the PTUI group, the 86 women were subdivided into the balloon group that had PBOIIA (group A1; *n* = 58) and the control group that did not have PBOIIA (group B1; *n* = 28). In the non-PTUI group, the 334 women were subdivided into the balloon group (group A2; *n* = 190) and the control group (group B2; *n* = 144).

Comprehensive management of patients with placenta previa and accreta required the cooperation of a multidisciplinary medical team in our hospital. All included patients should perform scheduled CS at 35–36 + 6 weeks of gestation [11], while some of patients may have been at a later gestational age when they were transferred to our center, and we performed a scheduled cesarean as soon as possible. Fluid transfusion, blood transfusion, strong uterine contraction drugs, and conservative surgical treatments were provided to patients during CS. If the conservative treatments were not effective, hysterectomy was performed to save the patient’s life. Since the effectiveness of PBOIIA in patients with placenta previa and accreta remains controversial and it is not definitively reliable, and there are complications associated with PBOIIA. The multidisciplinary team conducted a thorough discussion and evaluation of each patient’s condition and imaging indicators before the operation, and confirmed that the patients with placenta previa and accreta had the indications of placement of PBAIIO. The final decision of preoperative prophylactic placement of balloon occlusion was jointly made by the surgeon and patient after full communicating and informing about the pros and cons of balloon placement. All patients who accepted PBOIIA provided written informed consent, and balloon occlusion of the right and left internal iliac arteries was inflated in all cases.

### Placement of the occlusion balloon

**Fig. 2:**
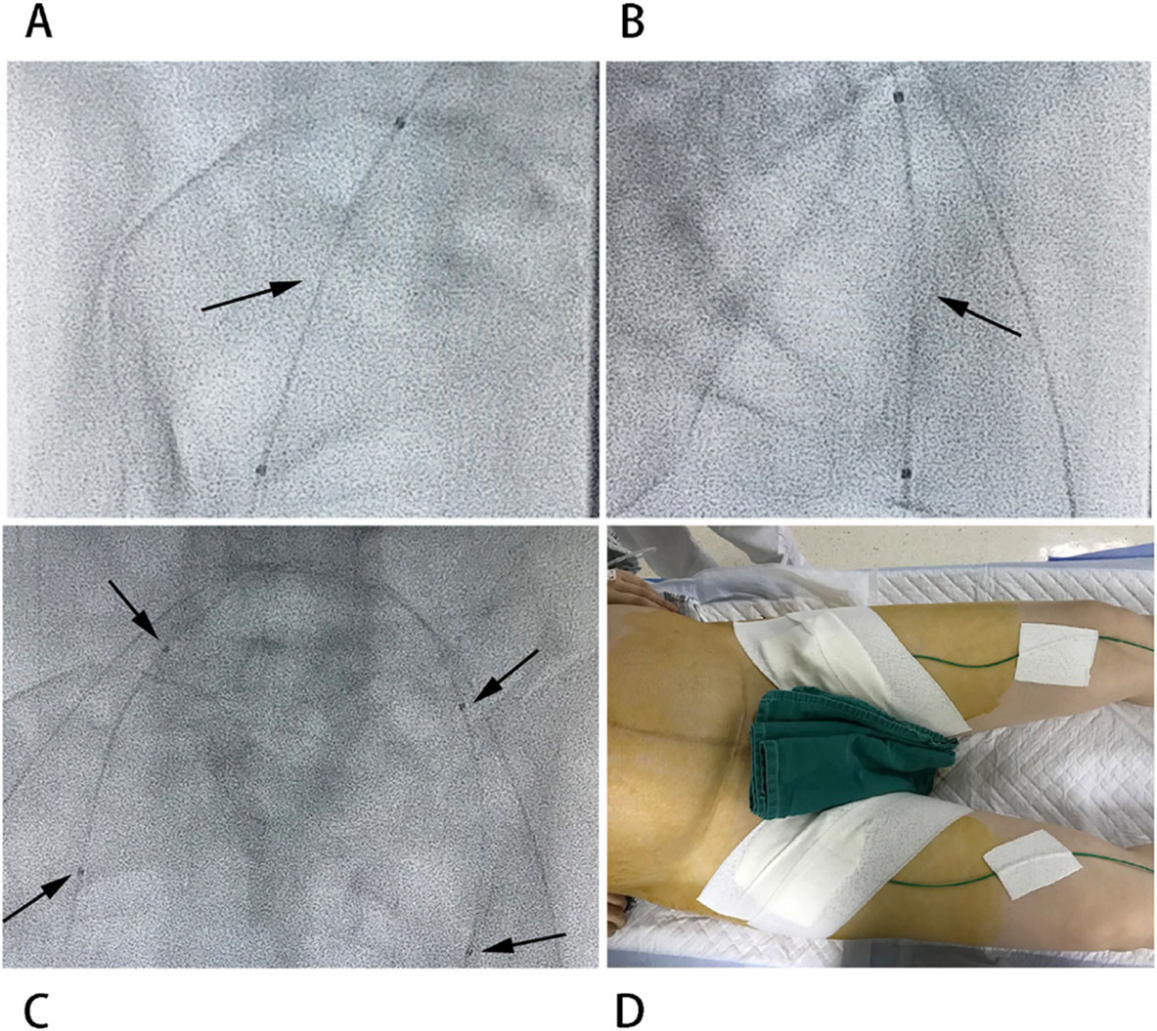
Angiographic image of occlusion balloons placed within the internal iliac arteries. **A** balloon placed within the right internal iliac artery (allow); **B** balloon placed within the left internal iliac artery (allow); **C** A panoramic view of prophylactic balloon occlusion of internal iliac arteries: The position between the two arrows is the balloon (allows); **D** posture of patient with PBOIIA

In the balloon group, all included pregnant women were fully informed of the benefits and complications of PBOIIA by their doctors before CS. For the surgical procedure, after routine disinfection and laying of towels, bilateral femoral arteries were punctured by the Seldinger technique and a 5-French vascular sheath was inserted. A 5-French Cobra and 0.035-in. guidewire were used to guide the balloon catheter into the bilateral internal iliac artery through the vascular sheath, with the balloon catheter tip slightly above the opening of the bilateral uterine artery (low-profile PTA balloon dilatation catheter PTA5–35–80-8-6.0; Cook Medical Inc., Bloomington, IN, USA). An indwelling catheter was fixed in both lower limbs and the patients were brought into the operating room. During the operation, approximately 2 mL of eunepiac, which is a diluent contrast agent, was injected to temporarily inflate the internal iliac artery balloon after the fetus was delivered and the umbilical cord was cut. This diluent contrast agent can block blood flow of bilateral internal iliac arteries and reduce the amount of uterine bleeding [12], while the balloon will be released for 5 min every 20–30 min to ensure the patient’s normal lower extremity circulation, and avoid rupture and bleeding of the collateral circulation vessels (Fig. 2).

### Clinical characteristics and outcomes

The clinical characteristics of the included patients were collected by reviewing the medical records. The clinical indicators were retrospectively collected by two obstetricians, and if any discrepancy existed, it was resolved by a third obstetrician. All reviewers were blinded to the selection of therapy and surgery conditions of patients. The clinical indicators included maternal and neonatal characteristics, perioperative blood indicators, and surgical outcomes during hospitalization. Maternal and neonatal characteristics included maternal age, gravidity, parity, number of previous CSs and abortions, gestational age at delivery, body mass index (BMI), birth weight of the newborn, 1-min Apgar score, neonatal intensive care unit (ICU) admission, neonatal asphyxia, and neonatal death. Ultrasound characteristics included cervical canal length, thickness of the placenta above the cervix, thickness of the placenta in the lower uterine segment, placenta position, myometrial thinning, multiple placental sinusoids, dilation of the cervical canal, loss of retroplacental clear zone, abnormal blood flow in retroplacental space, bladder wall interruption. Perioperative blood indicators included hemoglobin (HGB), hematocrit, platelets, prothrombin time, activated partial thromboplastin time, and fibrinogen. Surgical outcomes included intraoperative EBL, cesarean hysterectomy, packed red blood cell (PRBC) transfusion (in China, one unit of PRBC is approximately equal to 200 mL of whole blood), fresh frozen plasma transfusion, platelet transfusion, the volume of autologous blood transfusion, blood loss within 24 h postoperatively, maternal ICU admission rate, postoperative pyrexia (≥38.5 °C), anemia (HGB < 100 g/L), total hospitalization costs, and surgery costs. Among the clinical outcomes, intraoperative EBL, cesarean hysterectomy rate and blood transfusion are the primary outcomes of this study.

### Statistical analysis

Categorical variables are presented as number/proportion (%) and were analyzed by the chi-square test. The Kolmogorov–Smirnov test was performed to determine the normality of continuous variables. Data are shown as mean ± standard for normally distributed variables. If variables were normally distributed, the independent t-test was used for analysis. The Mann–Whitney U test was used for the analysis of non-normally distributed data and data are shown as the median (range). All statistical analyses and data processing was conducted by SPSS18.0 statistical software (IBM, Armonk, NY, USA). *P* values and 95% confidence intervals are shown for assessment of clinical indictors of patients with placenta previa and accreta. *P* < 0.05 indicates that the difference was significant.

## Results

### Characteristics of trial participants characteristics between balloon and control groups

The mean maternal age in the balloon group and control group was 32.48 ± 5.09 and 32.70 ± 4.72 years, respectively, the median values (range) of previous CSs were 1 (1–3) and 1 (1–3) in the balloon group and control group, and the median values (range) of gestational age at delivery in the balloon and control group were 36^+ 3^(28^+ 3^–40^+ 4^) weeks and 36^+ 2^(28^+ 1^–39^+ 3^) weeks respectively. There were no significant differences in maternal basic characteristics, such as maternal age, gravidity, parity, number of previous CSs and abortions, gestational age at delivery, and body mass index, between the balloon and control groups, which indicated that the two groups were comparable. With regards to neonatal characteristics, the newborn weight of the balloon group was significantly higher and the rate of neonatal ICU admission was lower than those in the control group (both *P* < 0.05). There were no differences in the neonatal asphyxia rate, neonatal death rate, and 1-min Apgar score between the balloon and control groups. Moreover, there were no significant differences in ultrasound indicators between the balloon and control groups (*P* >0.05). Baseline and ultrasound characteristics were comparable between two groups (Table 1).

**Table 1:** Characteristics of trial participants characteristics between balloon and control groups

Indicators	Balloon group	Control group	*P*	t/z/x^2^
	*n*= 248	*n* = 172		
Maternal characteristics				
Maternal age (years)	32.5 ± 5.1	32.7 ± 4.7	0.654	0.448
BMI (kg/m^2^)	21.7 ± 2.7	21.2 ± 2.5	0.081	−1.744
Gravidity (n)	4 (2–13)	4 (2–10)	0.539	−0.615
Parity (n)	1 (1–5)	1 (1–3)	0.123	−1.543
Number of previous CSs (n)	1 (1–3)	1 (1–3)	0.332	−0.971
Number of previous abortions (n)	2 (0–7)	2 (0–8)	0.531	−0.626
Gestational age at delivery (weeks)	36^+ 3^ (28^+ 3^–40^+ 4^)	36^+ 2^ (28^+ 1^–39^+ 3^)	0.137	−1.487
Neonatal characteristics				
Birthweight of the newborn (g)	2778.3 ± 410.6	2592.7 ± 477.4	0.000	−4.041
1 min Apgar score	9.1 ± 1.6	9.0 ± 1.8	0.696	−0.399
Neonatal ICU admission (n%)	50/248 (20.16%)	55/172 (31.98%)	0.006	7.562
Neonatal asphyxia (n%)	34/248 (13.71%)	26/172 (15.12%)	0.685	0.164
Neonatal death (n%)	2/248 (0.81%)	4/172 (2.33%)	0.197	1.664
Ultrasound indicators				
Cervical canal length	2.89 ± 0.09	3.06 ± 0.05	0.116	1.578
Thickness of the placenta above the cervix	3.91 ± 0.20	3.55 ± 0.15	0.139	−1.468
Thickness of the placenta in the lower uterine segment	3.41 ± 0.13	3.35 ± 0.11	0.664	- 0.435
Anterior placenta	224 (90.3%)	151 (87.8%)	0.409	0.681
Myometrial thinning	169 (68.1%)	112 (65.1%)	0.421	0.517
Multiple placental sinusoids	206 (83.1%)	134 (77.9%)	0.186	1.752
Dilation of the cervical canal	71 (28.6%)	40 (23.3%)	0.219	1.508
Loss of retroplacental clear zone	204 (82.3%)	132 (76.7%)	0.165	1.930
Abnormal blood flow in retroplacental space	235 (94.8%)	158 (91.9%)	0.234	1.418
Bladder wall interruption	42 (16.9%)	23 (13.3%)	0.321	0.986

Note-Data are presented as mean ± SD, as median (Range) or as number (%)

*BMI* - body mass index; *CS* - cesarean section; *ICU* - intensive care unit

### Comparison of perioperative blood indicators between the balloon and control groups

There were no significant differences in preoperative blood indicators and postoperative blood indicators, including HGB, hematocrit, platelets, prothrombin time, activated partial thromboplastin time, and fibrinogen, between the balloon and control groups (*P* >0.05) (Table 2).

**Table 2:** The comparison of perioperative blood indicators between balloon group and control groups

Blood Indicators		Balloon Group	Control Group	*P*	t/z
		(*n* = 248)	(*n* = 172)		
HGB (g/L)	Preoperative	110.2 ± 13.8	109.2 ± 14.4	0.468	−0.727
	Postoperative	95.1 ± 13.4	95.4 ± 15.8	0.818	0.23
HCT (%)	Preoperative	33.4 ± 3.6	33.1 ± 3.8	0.419	−0.809
	Postoperative	28.5 ± 3.9	28.5 ± 4.6	0.87	−0.164
PLT (10^9^/L)	Preoperative	157.0 (58.0–344.0)	168.0 (57.0–365.0)	0.28	−1.081
	Postoperative	135.0 (39.0–339.0)	134.5 (26.0–300.0)	0.982	−0.023
PT (s)	Preoperative	11.9 (10.2–14.6)	12.0 (10.2–13.7)	0.279	−1.082
	Postoperative	12.4 (10.8–14.5)	12.3 (10.8–14.4)	0.644	−0.462
APTT (s)	Preoperative	28.4 (12.4–45.7)	28.2 (19.9–40.2)	0.452	−2.774
	Postoperative	32.3 (20.1–45.3)	31.4 (22.0–45.2)	0.296	−1.045
FIG (mg/dL)	Preoperative	384.5 (167–666)	385.0 (79.0–795.0)	0.938	−0.078
	Postoperative	326.0 (161.0–701.0)	341.0 (154.0–641.0)	0.269	−1.106

Note–Data are presented as mean ± SD, or as median (Range)

*HGB* hemoglobin; *HCT* hematocrit; *PLT* platelet; *PT* prothrombin time,

*APTT* activated partial thromboplastin time; *FIG* fibrinogen

### Comparison of surgical outcomes between the balloon and control groups

Median blood loss and the cesarean hysterectomy rate were not significantly different between the balloon and control groups, the median blood loss was 2200 mL (range, 500–12,000) in the balloon group and 2150 mL (range, 500–15,000) in the control group, and in balloon group, 36.3% patients with cesarean hysterectomy versus 35.5% in control group, which were no significant difference (*P* >0.05). The amount of PRBC transfusion, PLT transfusion and the rate of maternal ICU admission were significantly lower in the balloon group than in the control group (*P* < 0.05). Moreover, we re-calculated the rate between two groups in amount of PRBCs > 4 units and 8 units, and found that there was no statistical difference in the proportion of PRBCs > 4 units between the two groups, however, the proportion of PRBCs>8 units in balloon group was significantly lower in compare to the control group. The total hospitalization and surgery costs in the balloon group were significantly higher than those in the control group (45,624.4 ± 11,061.9 verus 37,523.1 ± 14,662.2, CYN and 19,910.6 ± 2622.6 verus 11,850.5 ± 3146.1, CYN, both *P* < 0.05). However, there were no significant differences in FFP transfusion, volume of autologous blood transfusion, blood loss within postoperative 24 h, operative fluids transfusion, postoperative length of stay, postoperative pyrexia (≥38.5 °C), anemia (HGB < 100 g/L) and urologic complications (bladder injury, ureter injury and vesico vaginal fistula) between the balloon and control groups (*P* > 0.05) (Table 3).

**Table 3:** Comparison of surgical outcomes of 420 patients with placenta previa and accreta

Indicators	Balloon Group	Control Group	*P*	t/z/x^2^
	(*n* = 248)	(*n* = 172)		
EBL (ml)	2200 (500–12,000)	2150 (500–15,000)	0.897	−0.13
PRBCs transfusion (U)	3 (0–31.5)	3 (0–39)	0.042	−2.037
PRBCs > 4 units transfused	73/248	65/172	0.073	3.214
PRBCs > 8 units transfused	28/248	40/172	0.001	10.716
FFP transfusion (U)	0 (0–2250)	0 (0–2800)	0.171	−1.37
PLT transfusion (U)	0 (0–1)	0 (0–1)	0.035	−2.104
Autologous blood transfusion (ml)	220 (0–2700)	217 (0–2144)	0.527	−0.633
Operative fluids transfusion (ml)	4000 (1000–15,600)	3700 (1700–17,700)	0.137	−1.487
Blood loss within postoperative 24 h (ml)	35 (0–2210)	40 (0–1908)	0.555	−0.590
Postoperative length of stay (d)	5 (2–17)	5 (2–16)	0.497	−0.68
Maternal ICU admission of days (d)	2 (1–5)	2 (1–6)	0.310	−1.015
Total hospitalization costs (CYN)	45,624.4 ± 11,061.9	37,523.1 ± 14,662.2	0.000	−6.074
Surgery costs (CYN)	19,910.6 ± 2622.6	11,850.5 ± 3146.1	0.000	−27.328
Maternal ICU admission rate (n%)	42/248 (16.94%)	47/172 (27.33%)	0.010	6.565
Postoperative pyrexia (≥38.5 °C) n%	24/248 (9.68%)	10/172 (5.81%)	0.153	2.038
Postoperative anemia (HGB < 100 g/L), n%	158/248 (63.71%)	103/172 (59.88%)	0.427	0.632
Cesarean hysterectomy (n%)	90/248 (36.3%)	61/172 (35.5%)	0.862	0.03
Bladder injury (%)	8/248 (3.23%)	5/172 (2.91%)	0.853	0.034
Ureter injury	6/248 (2.42%)	3/172 (1.74%)	0.638	0.221
Vesico vaginal fistula	0/248	0/172	/	/

Note-Data are presented as mean ± SD, as median (Range) or as number (%)

EBL: estimated blood loss; PRBCs: packed red blood cells; FFP: fresh frozen plasma; PLT: platelet; ICU: intensive care unit

### Comparison of intraoperative conditions in the PTUI subgroup

We performed subgroup analysis on whether PTUI surgery was performed in the balloon and control groups. There was no significant difference in EBL [2200 (900–5500)] verus 2200 (500–12,000), *P* > 0.05] in patients of balloon group between Group A1 (PTUI surgery) and Group A2 (non-PTUI surgery), and there was also no significant difference in EBL [2200 (1000–6000) verus 2100 (500–15,000) ml, *P* > 0.05] in patients of control group between Group B1 (PTUI surgery) and Group B2 (non-PTUI surgery). The hysterectomy rates of the PTUI surgery group (Group A1 and Group B1) were significantly lower than those of the non-PTUI surgery group (Group A2 and Group B2) [(5.17% verus 45.79%), (7.14% verus 40.97%); *P* < 0.05] (Tables 4 and 5).

**Table 4:** Comparison of EBL and cesarean hysterectomy rate in Group A1 and A2 of balloon group

Indicators	Group A1	Group A2	*P*	z/x^2^
	PTUI surgery (*n* = 58)	non-PTUI surgery (*n* = 190)		
EBL (ml)	2200 (900–5500)	2200 (500–12,000)	0.456	*z* = −0.745
Cesarean hysterectomy (n%)	3/58 (5.17%)	87/190 (45.79%)	0.000	*x*^*2*^ = 31.707

Note-Data are presented as mean ± SD, or as number (%)

*PTUI* parallel transverse uterine incision; *EBL* estimated blood loss

**Table 5:** Comparison of EBL and cesarean hysterectomy rate in Group B1 and B2 of control group

Indicators	Group B1	Group B2	*P*	t/x2
	PTUI surgery (*n* = 28)	non-PTUI surgery (*n* = 144)		
EBL (ml)	2200 (1000–6000)	2100 (500–15,000)	0.771	*z* = − 0.291
Cesarean hysterectomy (n%)	2/28 (7.14%)	59/144 (40.97%)	0.001	*x*^*2*^ = 11.72

Note-Data are presented as mean ± SD, or as number (%)

*PTUI* parallel transverse uterine incision; *EBL* estimated blood loss

## Discussion

Placenta previa and accreta after previous CS are extremely serious obstetric conditions associated with a high risk of intraoperative massive hemorrhage. These conditions dramatically increase the risk of blood transfusion and hysterectomy during CS [13, 14]. Placenta previa and placenta accreta are the two major risk factors for postpartum hemorrhage [15]. The incidence of placenta previa after CS is 1.22% [16]. However, prior CS and placenta previa appear to be the major risk factors for placenta accreta. Previous studies have reported that the incidence of placenta accreta was 3.3, 11, 40, 61, 67, and 67% after first, second, third, fourth, fifth, and sixth or more cesarean deliveries respectively in pregnant women with placenta previa [11, 17].

Owing to the high morbidity associated with placenta previa and accreta, accurate preoperative diagnosis and a multidisciplinary medical team for management of these conditions play a vital role [4, 18]. Prenatal ultrasound and MRI techniques have been used to diagnose and guide clinical management, and favorable outcomes have been achieved [19, 20]. With the wide use of ultrasound and MRI in the medical field, abnormal placenta accreta can be diagnosed in advance, and a treatment plan can be provided for reducing intraoperative blood loss. Surgical hemostasis and uterine contractile agents are used to manage intraoperative hemorrhage and preserve the uterus during CS. If all methods of hemostasis fail to control the bleeding, hysterectomy is the ultimate solution for patients with placenta previa and accrete [21].

In recent years, an increasing amount of hemostasis methods have been applied in CS, such as the prophylactic intravascular balloon occlusion technique. As early as 1997, PBOIIA was first reported as a hemostatic method in CS for patients with placenta percreta [22]. With the increasing use of PBOIIA in CS for treating placenta previa and accreta, which plays an important role in managing uncontrolled hemorrhage for obstetricians and anesthetists. However, the efficacy of PBOIIA reported in the literature is controversial.

Some scholars reported that PBOIIA was an effective hemostasis method in CS. Fan et al. conducted a prospective observational study, which included 163 patients with placenta previa and accreta., and they found PBOIIA was an effective strategy for controlling severe hemorrhage, which can effectively reduce the amount of intraoperative blood loss for patients with placenta previa–accreta (1236.0 verus 1694.0 mL) [21]. Picel et al. reported that, in 151 patients with invasive placenta undergoing cesarean hysterectomy, there was a significant difference in blood loss (2000 verus 2500 mL) and PRBC transfusions (2 verus 5 U) in the balloon group compared with the control group [23]. Zhou et al. studied 83 patients with pernicious placenta previa coexisting with placenta accreta and found that PBOIIA was an effective method for managing postpartum hemorrhage [24]. These findings indicated that PBOIIA could reduce intraoperative blood loss and transfusion requirements. However, some researchers failed to show that PBOIIA improves maternal outcomes. Previous randomized, controlled trials included 13 patients who were diagnosed with placenta accreta in the intervention group and 14 cases in the control group found that there were no significant differences in calculated blood loss (4950 verus 4709 mL) and PRBC units transfused (5.2 verus 4.1 U) between the intervention and control groups [25, 26]. Chen et al. compared 83 patients with placenta previa and accreta who underwent cesarean hysterectomy in the balloon group and 31 patients in the control group, they found PBOIIA had no significant effects on reducing EBL (3000 vs 3700 mL) and improving maternal outcomes [27]. These findings suggested that PBOIIA had no benefit in patients with placenta accreta.

Plenty of studies have investigated the effect of PBOIIA, but the sample sizes were small and the power of the studies was limited. We conducted a large-sample study to evaluate the effectiveness and practicality of PBOIIA for patients with placenta previa and accreta. In our study, we included 420 patients with placenta previa and accreta, and divided them into the balloon (248 cases) and control groups (172 cases). The results of this study showed that PBOIIA could reduce the rate of intraoperative PRBCs>8 units (11.29% verus 23.26%), but it has no benefit in improving maternal outcomes of reducing intraoperative blood loss [2200 (500–12,000) verus 2150 (500–15,000)] and hysterectomy rates((36.3% verus 35.5%), which are consistent with the previous negative finding that PBOIIA had no significant effects on improving maternal outcomes. In clinical practice, it was found that the application of PBOIIA could reduce the intraoperative bleeding rate of patients to a certain extent, and then the surgeons and anesthesiologists could more accurately assess the amount of blood loss and achieve reasonable blood transfusion for patients taking the PBOIIA. thereforeo a more higher blood transfusion volume in the control group than that in the balloon group, even though there was no difference in blood loss, eventually leading to the inconsistency of intraoperative blood loss and blood transfusion between the two groups.

Among the included patients, some underwent PTUI surgery simultaneously. A previous study showed that PTUI had a significant effect on reducing intraoperative blood loss and the hysterectomy rate [10]. Therefore, we conducted subgroup analysis to examine the effectiveness of PBOIIA when eliminating the effect of PTUI surgery during CS. We found that there were no significant differences in intraoperative EBL, but the rate of hysterectomy was significant decreased when PTUI surgery was taken. Additionally, no significant differences in postoperative blood indicators were found between the two groups.

This finding indicated that PBOIIA may have no beneficial effect on recovery of the blood indicators in patients after CS. However, the total hospitalization cost and surgery costs in the balloon group were significantly higher than those in the control group, which greatly increased the financial burden of patients. Therefore, we should take a cautious attitude towards PBOIIA according to the individual situation of the patients. Abdominal aorta artery balloon occlusion is more effective than internal iliac artery occlusion in hemostasis in patients with placenta previa and accreta, but it is associated with a higher risk of vascular-related complications [5]. The indications of abdominal aorta artery balloon occlusion should be strictly controlled in clinical practice. Further studies are required to compare the advantages and disadvantages of the two types of intervention surgery in patients with placenta previa and accreta [5, 28].

Balloon occlusion-related complications are rarely reported, with a rate of approximately 6–15.8%, and these include ischemia, thrombosis, pain, fever, anemia, hematoma, and infection [29, 30]. In our study, there were no significant differences in the rates of postoperative fever (≥38.5 °C) and anemia (HGB < 100 g/L). There were also no balloon occlusion-related complications in patients after major surgery in our study, such as ischemia and thrombosis.

### Strengths and limitations

The main advantage of our study is that the number of included patients was larger than that in previous studies, and therefore, the results are more reliable. We also performed subgroup analysis to evaluate the effectiveness of PTUI surgery after eliminating the effect of PBOIIA on hemorrhage, further confirm the efficacy of PTUI surgery and provide a more reliable analysis of the results. Our study also bears some limitation. Firstly, our study is a retrospective, single-center study. Additionally, our study was observational, and the included subjects could not be randomly allocated. Therefore, the selection bias may have been present. However, we calculated the basic characteristics and preoperative imaging indicators of the two groups, and the demographic of the two selected groups were matched.

## Conclusion

Our study shows that PBOIIA could reduce the intraoperative PRBCs in patients with massive hemorrhage, but it has no benefit in improving maternal outcomes of reducing intraoperative blood loss and hysterectomy rates for patients with placenta previa and accreta, additionally, PBOIIA significantly increased the financial burden of patients. When the effect of PTUI surgery on hemorrhage is eliminated, there is still no significant difference in intraoperative EBL and the hysterectomy rate. Therefore, PBOIIA should not be routinely recommended to patients with placenta previa and accreta.

## Data Availability

The datasets used and/or analyzed during the current study are available from the corresponding author on reasonable request.

## Abbreviations

PBOIIA: prophylactic balloon occlusion of the internal iliac arteries
CS: cesarean section
PTUI: parallel transverse uterine incision
EBL: estimated blood loss
